# Reflectance Confocal Microscopy Can Help in Detecting Clinically Silent Mammary Paget’s Disease

**DOI:** 10.3390/diagnostics14232717

**Published:** 2024-12-03

**Authors:** Carmen Cantisani, Alberto Taliano, Caterina Longo, Stefano Astorino, Vito Gomes, Gianluca Caruso, Mariano Suppa, Stefania Guida, Anna Pogorzelska-Antkowiak, Giovanni Pellacani

**Affiliations:** 1 UOC of Dermatology, Policlinico Umberto I Hospital, Sapienza Medical School of Rome, 00161 Rome, Italy; albertotaliano1@gmail.com (A.T.); giovanni.pellacani@uniroma1.it (G.P.); 2 Department of Dermatology, University of Modena and Reggio Emilia, 41121 Modena, Italy; caterina.longo@unimore.it; 3 Azienda Unità Sanitaria Locale-IRCCS di Reggio Emilia, Skin Cancer Center, 42122 Reggio Emilia, Italy; 4 Unit of Dermatology and Venereology, Celio Military Hospital, 00100 Rome, Italy; stefano.astorino@virgilio.it; 5 Unit of Cellular Pathology, San Filippo Neri Hospital, 00135 Rome, Italy; semog@libero.it (V.G.); gianluca.caruso79@gmail.com (G.C.); 6 Department of Dermatology, Hôpital Erasme–Hôpitaux Universitaires de Bruxelles (HUB), 1000 Brussels, Belgium; dr.marianosuppa@gmail.com; 7 School of Medicine, Vita-Salute San Raffaele University, 20132 Milan, Italy; drstefaniaguida@gmail.com; 8 Dermatology Clinic, IRCSS San Raffaele Hospital, 21132 Milan, Italy; 9 EsteDerm Private Dermatology Clinic, 43-100 Tychy, Poland; annaporzelska03@wp.pl

**Keywords:** dermoscopy, reflectance confocal microscopy, Paget’s disease, skin lesion

## Abstract

Early detection and comprehensive diagnostic approaches for breast cancer are essential for improving prognosis. When it comes to changes in the skin of the breast or the nipple–areola complex (NAC), particularly if they are unilateral, it is essential to be vigilant, as these changes could be an early sign of underlying malignancy or other pathologies. Primary breast malignancies, such as mammary Paget’s disease (MPD), can manifest as erythema, scaling, or ulceration of the NAC, while secondary cutaneous metastases from other breast carcinomas may present as nodules, erythematous plaques, or inflammatory reactions. Non-malignant inflammatory conditions, including eczema or mastitis, can also mimic these changes; histologic evaluation is the gold-standard diagnostic tool. The usefulness of conventional diagnostic techniques breast lesions has been confirmed, but in recent years, reflectance confocal microscopy (RCM) and optical coherence tomography (OCT) have emerged as additional tools to diagnose cases characterized by cutaneous changes; they may, therefore, result in new perspectives on the non-invasive diagnosis of MPD. RCM is a non-invasive diagnostic technique that allows high-resolution images of the skin at microscopic level in real time, offering a promising approach to the non-invasive diagnosis of MPD, particularly when a lesion is not clinically evident and may mimic other benign or inflammatory conditions. We describe an atypical clinical presentation of mammary Paget’s disease diagnosed early by reflectance confocal microscopy evaluation and confirmed histologically.

**Figure 1:**
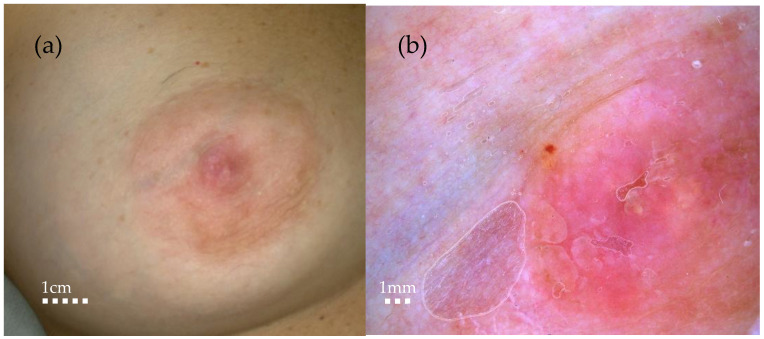
(**a**) Macroscopic aspect. (**b**) Video-dermoscopy aspect showing mild erythema and few yellowish scales. A lady in her 40s presented to our clinic complaining about itching around her right nipple: there was no personal or familiar history of neoplasia. Breast cancer remains a significant health concern worldwide, necessitating early detection and treatment. The rapid appearance of or a change in the shape of skin lesions on the breast or just the nipple–areola complex, especially if it is unilateral, needs to be carefully evaluated [1]. The differential diagnosis of lesions involving the nipple and areola complex encompasses a broad spectrum of conditions, ranging from inflammatory diseases to benign and malignant tumours, and is thus a challenging process [2].

**Figure 2:**
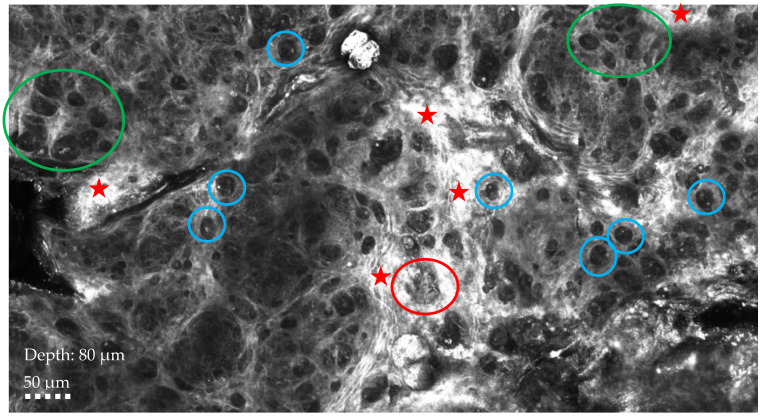
RCM showing chaotic architecture at the dermo-epidermal junction, characterized by the presence of single atypical cells appearing as dark holes with bright particles (blue circle), big tumor nests (red circle), and highly reflective tumor nests (green circle) surrounded by severe inflammation (red star). Ultrasound, mammography, or magnetic resonance imaging (MRI) are the most used non-invasive diagnostic techniques for breast cancer, but in recent years, reflectance confocal microscopy and optical coherence tomography have emerged as additional tools to diagnose cases characterized by cutaneous changes [3,4,5]. RCM is a non-invasive diagnostic technique that allows high-resolution images of the skin to be taken at a microscopic level in real time. It is non-invasive, painless, and repeatable, making it a valuable tool in the early diagnosis and monitoring of skin diseases. MPD represents a significant diagnostic and therapeutic challenge, usually being detected only at later stages; therefore, the recent use of RCM is gaining attention due to its ability to identify in vivo typical features of Paget’s disease even when lesions are not yet clinically suggestive, playing a role in the differential diagnosis between MPD and nipple eczema, two conditions that often present with similar clinical manifestations. However, RCM application in MPD presents certain limitations that hinder its widespread use: one of the primary challenges is the tissue heterogeneity observed in MPD lesions, where superficial epidermal involvement can complicate the interpretation of confocal images. The technique’s inability to penetrate deeply into thickened or hyperkeratotic skin can limit its capacity to fully visualize the extent of intraepidermal carcinoma cells, which are characteristic of Paget’s disease. Furthermore, another challenge is the limited diffusion and availability of this technology, which restricts its widespread clinical use, particularly in settings lacking specialized equipment or expertise. As a result, reflectance confocal microscopy is often not a viable option for routine diagnostic purposes in many clinical environments. In addition, MPD presents with heterogeneous clinical and histopathological features, and current imaging techniques, including RCM, lack universally accepted guidelines and diagnostic criteria. This variability can lead to diagnostic uncertainty, limiting the role of reflectance confocal microscopy as a standalone tool. Despite these challenges, RCM remains a promising adjunctive technique for the real-time imaging of MPD, particularly in terms of detecting superficial epithelial changes. Further technological advancements may help mitigate these current limitations: the synergistic application of non-invasive diagnostic techniques holds promise as a comprehensive diagnostic tool for skin breast lesion evaluation, offering real-time, non-invasive imaging with potential implications in clinical practice of improved diagnosis, therapeutic decisions, and treatment monitoring. It can also help to achieve optimal aesthetic outcomes, significantly improving patients’ quality of life and optimizing clinical outcomes.

**Figure 3:**
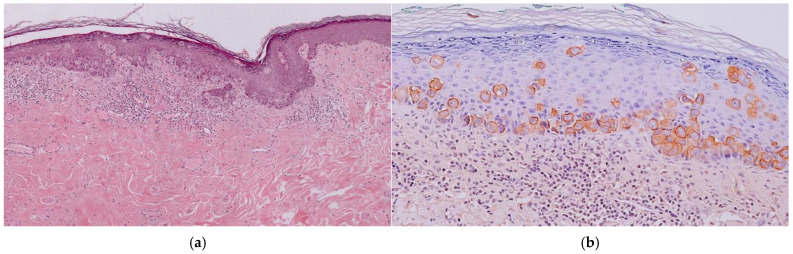
(**a**) Histological aspect: Infiltration of the epidermis by large and atypical epithelioid cells. (**b**) Positive immunohistochemical staining for cytokeratin 7.

## Data Availability

Not applicable.
